# Image harmonization and deep learning automated classification of plus disease in retinopathy of prematurity

**DOI:** 10.1117/1.JMI.10.6.061107

**Published:** 2023-10-03

**Authors:** Ananya Subramaniam, Faruk Orge, Michael Douglass, Basak Can, Guillermo Monteoliva, Evelin Fried, Vanina Schbib, Gabriela Saidman, Brenda Peña, Soledad Ulacia, Pedro Acevedo, Andrew M. Rollins, David L. Wilson

**Affiliations:** a Case Western Reserve University, Department of Biomedical Engineering, Cleveland, Ohio, United States; b Case Medical Center University Hospitals, Department of Ophthalmology, Cleveland, Ohio, United States; c Hospital Italiano de San Justo Agustin Rocca, Buenos Aires, Argentina; d Hospital de Niños Sor Maria Ludovica, Buenos Aires, Argentina; e Hospital Evita Pueblo de Berazategui, Buenos Aires, Argentina; f Centro Integral de Salud Visual Daponte, Buenos Aires, Argentina; g Mineserio de Salud Argentina, Ministry of Public Works Building, Buenos Aires, Argentina; h Fundación Oftalmológica Nacional, Bogotá, Colombia; i Case Western Reserve University, Department of Radiology, Cleveland, Ohio, United States

**Keywords:** retinopathy of prematurity, plus disease, fundus, image processing, deep learning

## Abstract

**Purpose:**

Retinopathy of prematurity (ROP) is a retinal vascular disease affecting premature infants that can culminate in blindness within days if not monitored and treated. A disease stage for scrutiny and administration of treatment within ROP is “plus disease” characterized by increased tortuosity and dilation of posterior retinal blood vessels. The monitoring of ROP occurs via routine imaging, typically using expensive instruments ($50 to $140 K) that are unavailable in low-resource settings at the point of care.

**Approach:**

As part of the smartphone-ROP program to enable referrals to expert physicians, fundus images are acquired using smartphone cameras and inexpensive lenses. We developed methods for artificial intelligence determination of plus disease, consisting of a preprocessing pipeline to enhance vessels and harmonize images followed by deep learning classification. A deep learning binary classifier (plus disease versus no plus disease) was developed using GoogLeNet.

**Results:**

Vessel contrast was enhanced by 90% after preprocessing as assessed by the contrast improvement index. In an image quality evaluation, preprocessed and original images were evaluated by pediatric ophthalmologists from the US and South America with years of experience diagnosing ROP and plus disease. All participating ophthalmologists agreed or strongly agreed that vessel visibility was improved with preprocessing. Using images from various smartphones, harmonized via preprocessing (e.g., vessel enhancement and size normalization) and augmented in physically reasonable ways (e.g., image rotation), we achieved an area under the ROC curve of 0.9754 for plus disease on a limited dataset.

**Conclusions:**

Promising results indicate the potential for developing algorithms and software to facilitate the usage of cell phone images for staging of plus disease.

## 1 Introduction

Retinopathy of prematurity (ROP), a disease affecting preterm infants characterized by abnormal development of retinal blood vessels, is the leading preventable cause of infantile blindness.^1^^,^^2^ Plus disease is an indicator of ROP severity and is characterized by severe dilation and tortuosity of retinal blood vessels in the posterior pole.^1^^,^^3^^,^^4^ Once plus disease is identified, infants are treated using laser photocoagulation or anti-vascular endothelial growth factor (anti-VEGF) injections with great success.^5^^,^^6^ However, an incorrect diagnosis or lack of screening can prove detrimental as the onset of plus disease can result in blindness within two weeks if left untreated, with nearly 50,000 infants blinded due to ROP annually worldwide.^7^^,^^8^ Routine retinal examination by skilled ophthalmologists is critical for early detection and treatment of ROP.

Screening for ROP is needed around the world. In high-income countries, routine screening protocols are well established, using expensive imaging systems costing 50 to 140K US dollars. As a result of screening, clinicians have managed to control ROP severity and rates of blindness.^9^^,^^10^ However, in middle and low-resource environments where access to adequate screening is limited and survival rates of premature infants have increased, ROP disease numbers have risen, resulting in the “third epidemic” of ROP.^10^ The first ROP epidemic occurred as a result of unrestricted use of oxygen, and the second was a result of increased premature infant survival rates in high-income countries.^11^ The rise of telemedicine has led to the use of smartphone cameras for routine imaging in low-resource environments, proving necessary as premature infantile survival rates increase, causing the relative number of ophthalmologists trained for ROP and plus disease diagnosis to decrease.^9^^,^^12^^,^^13^ Smartphones are advantageous in these settings due to their accessibility, low-cost, mobility, and access to the internet for referral. However, the image quality and field of view are much reduced compared with the expensive commercial systems.^12^ An advanced imaging device is the Natus RetCam (Middleton, Wisconsin, United States), a device costing ∼$140,000 US dollars. RetCam images have a 130 deg field of view, whereas the smartphone images have a field of view of ∼30  deg to 40 deg.^14^

There are reports of automated analysis of fundus images for ROP and plus disease diagnosis using images from the RetCam.^2^^,^^15^^,^^16^ Various works used large datasets (thousands of images) for model development consisting of standardized images with higher fields of view.^2^ Tan et al. used 4926 deidentified RetCam images to train inception-V3, a convolutional neural network (CNN), to diagnose the presence of plus disease with a resulting area under the ROC curve (AUC) of 0.993.^10^ Brown et al. employed two successive CNNs, one for vessel segmentation and the other for classification, to develop a high-accuracy model for three-stage plus disease classification.^2^ The success of such works encouraged us to apply similar methods to lower quality cell phone fundus images.

In this report, using cell phone images, we developed methods for image enhancement and harmonization as well as artificial intelligence (AI) algorithms for ROP disease staging. Images were obtained from collaborators across South America as part of the smartphone-ROP (SP-ROP) project using Volk diopter lenses and a variety of cell phone makes and models. This created a dataset with large variations in image quality, focus, lighting, and exposure, but we developed a preprocessing method to enhance vessels and regularize presentation to clinicians in all of the fundus images that we received. The same preprocessing method was used to prepare images for deep learning classification of plus disease. In this report, we describe preprocessing methods, image quality evaluations by physicians, deep learning classification of plus disease, and a comparison between AI classifications and those of expert physicians.

## 2 Images and Algorithms

### 2.1 Image Acquisition and Preprocessing

Methods for smartphone image acquisition are as follows. Collaborators in Argentina and Colombia acquired 440 images of the fundus of premature infants. Deidentified images were obtained from collaborating institutions under an approved Data Use Agreement. The Case Western Reserve University Institutional Review Board office deemed this project to be non-human subject research. These images were obtained as part of the SP-ROP project, which consists of physician representatives from various countries in South America, Central America, and North America. During image acquisition, physicians dilated the pupils of the infant using eye drops, placed either a Volk 28D or 20D lens over the eye, and acquired images using a smartphone camera. Smartphone camera makes and models varied. Deidentified data included ROP stage and plus disease level (“no plus,” “pre plus,” or “plus”), as determined in person by an expert physician using all clinical information available, including administered treatments. Images ranged in size from 300 to 1200 pixels in length and width and had variable lighting, sometimes with specular reflections. For each patient, physicians acquired images of both the central and peripheral view of the fundus. For the purposes of this study, we only used images that featured the optic nerve.

**Fig. 1:**
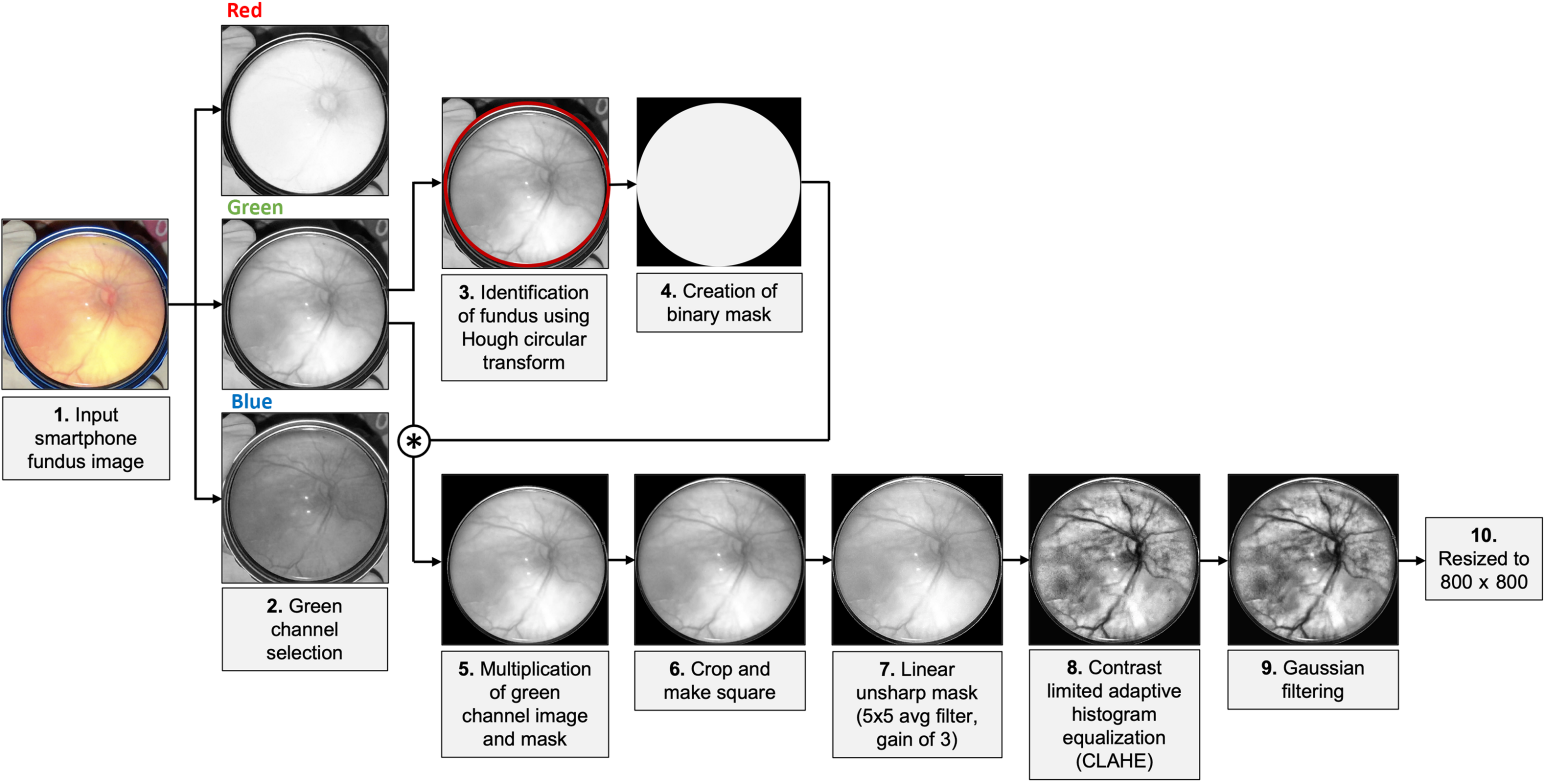
Preprocessing workflow with example image.

The image enhancement pipeline (Fig. 1) included various preprocessing steps and took into account variations in image size and orientation. We began by selecting the green channel of the RGB image, which has the greatest contrast between the vessels and fundus. A mask of the fundus region was identified using the Hough circular transform, an algorithm that detects circular structures within a given input range. The algorithm increments through edge points in an image, draws a circle with a center at the given edge point with radius r, and finds maxima in the coordinates of the perimeter of this circle. The circular structure identified was used to create a binary mask by which the green channel image was multiplied. The image was then cropped to include only the fundus and then made square via the addition of rows or columns of zero-valued pixels. Linear unsharp masking was then performed using a 5×5 averaging filter with a gain of 3. These values were qualitatively selected after testing a range of values on a subset of images with varying lighting and quality. This was followed by contrast limited adaptive histogram equalization, a version of adaptive histogram equalization that prevents noise enhancement and overamplification of contrast in uniform regions.^17^ Gaussian filtering was used to counteract any over-sharpening of non-vascular regions (i.e., specular reflection and artifacts) from previous steps. Images were finally resized to 800×800  pixels for uniformity.

### 2.2 Deep Learning Algorithms

The algorithms used in this study are explained below. Although plus disease has been stratified into three categories in recent years, the diagnosis of the “plus” category remains the indicator for administration of treatment.^18^ Therefore, we grouped the “no plus” image category with “pre plus” to develop a binary deep learning framework to distinguish these from images in the “plus” category. The ground truth diagnoses for our deep learning model were those obtained during image acquisition as part of a proper in-person clinical examination, during which physicians examined the infant using indirect binocular ophthalmoscopy, saw more than the isolated image (were able to achieve a larger field of view by moving the lens around the eye), and made treatment decisions based on their expanded evaluations.

Our binary deep learning model was developed using the GoogLeNet (inception-v1) network pretrained on the ImageNet dataset. The pretrained GoogLeNet is a 22-layer network that classifies images into 1000 object categories. To leverage this network, we froze the first 20 layers and replaced the last learnable layer and the final classification layer with layers relevant to our dataset. GoogLeNet was selected as the network of choice for transfer learning as it outperformed other networks pretrained on ImageNet, including Inception v3 and ResNet. Implementation on the untrained GoogLeNet was explored and abandoned, with poor results due to limited dataset size. We additionally investigated the impact of pretraining ImageNet-pretrained GoogLeNet on 5000 images from EyePACS, a large publicly available fundus image dataset for classification of diabetic retinopathy and fine tuning on our dataset. This yielded identical results on our held-out test set and was thus not included in our training workflow.

**Fig. 2:**
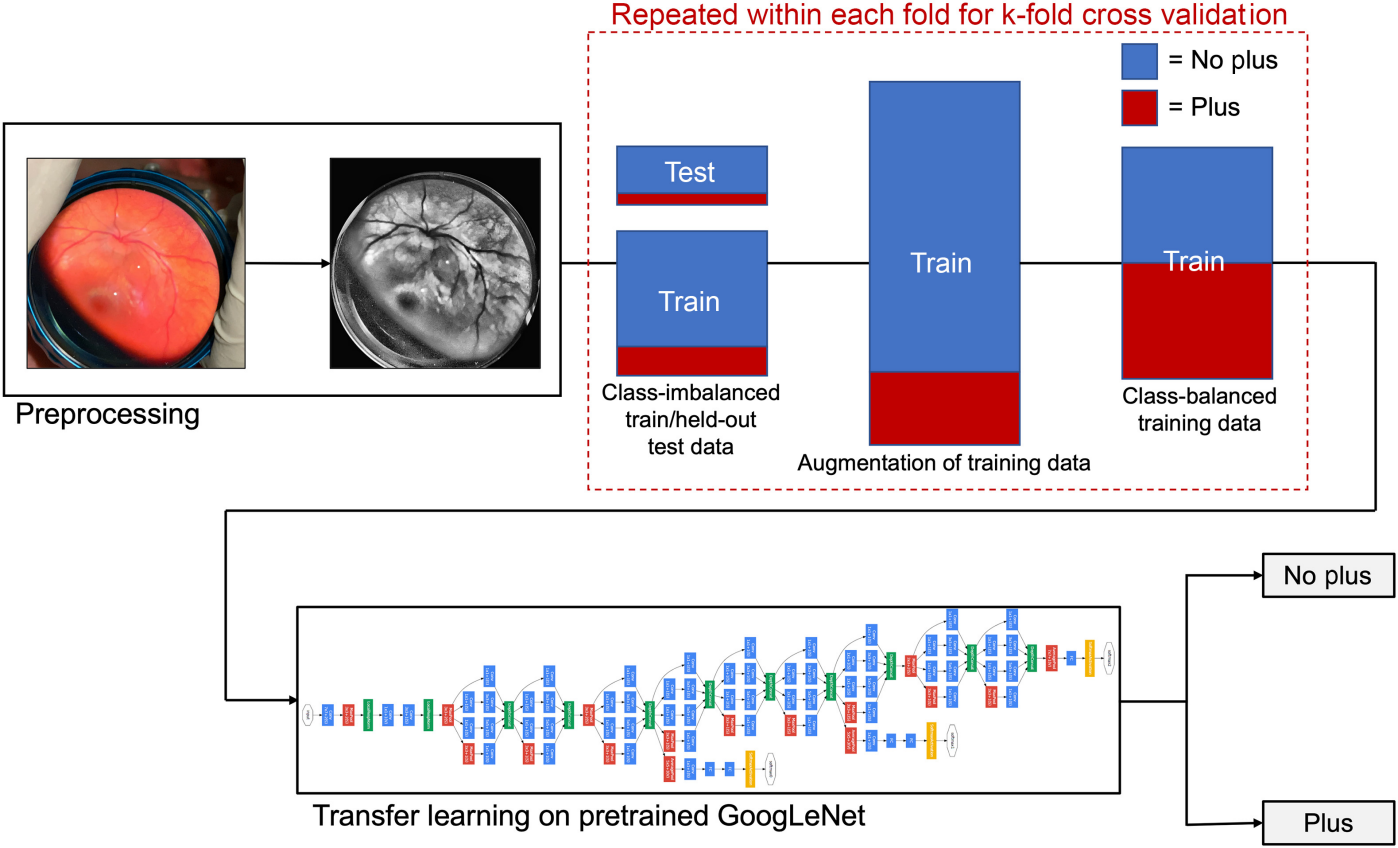
Deep learning workflow for binary classification and GoogLeNet (inception-v1) architecture.

Our dataset suffered from imbalance in the class distribution, with 385 “no plus” images and only 55 “plus” images. To mitigate the effects of class imbalance, we first performed large-scale augmentation on the training set, creating eight times each class type using five rotation augmentations, reflection over the x-axis, and reflection over the y-axis. Performing such transformations was physically reasonable as our original images had varied orientations depending on physician acquisition. From this augmented dataset, we randomly selected an equal number of images from each class. The network was trained on this balanced dataset and evaluated on a held-out unaugmented test data. This workflow, including the augmentation/randomized selection method to correct for class imbalance, is depicted in Fig. 2.

Given the nature of the disease, it is critical to consider the implications of unsuccessful classification. As such, we chose to employ the real-world-weight cross-entropy (RWWCE) loss function, a loss function that assigns costs to false negatives and false positives to incorporate information about the real-world impact of the problem.^19^ In the case of a false positive, the baby would receive an inaccurate plus disease diagnosis and would be treated via either anti-VEGF injections or laser photocoagulation.^20^^,^^21^ Although unlikely, along with either of these treatments come potential side effects, including loss of visual field, myopia, and retinal destruction.^20^ However, the cost of a false negative is much more detrimental, as an untreated case of plus disease can cause the baby to go blind within days.^7^ In terms of price point, treatment using anti-VEGF is 20,076 US dollars on average and treatment with laser photocoagulation is 17,129 US dollars.^22^ Estimates suggest the mean annual expenses per patient with blindness are US$ purchasing power parties (PPP) 14,882-24,180.^23^ It is important to note that this value is calculated for the US and may not be perfectly accurate for populations outside of the US as the cost of caretakers contributes to this estimate, which may not be accessible to all. To estimate the cost of blindness over a lifespan versus the cost of the procedure, we have selected a weight of $1,700,000 for false negatives and a weight of $17,000 for false positives (100:1). Recognizing that this is somewhat arbitrary, we tested the effect of changing this ratio to 50:1 and 200:1. The RWWCE loss for binary classification is calculated as

$$J_{\mathrm{brwwce}}=-\frac{1}{M}\overset{M}{\underset{m=1}{\sum }}[[w_{mcfn}\times y_{m}\times \log(h_{\theta }(x_{m}))+w_{mcfp}\times (1-y_{m})\times \log(1-h_{\theta }(x_{m})))],$$ (1)

where $J_{\mathrm{brwwce}}$ is the binary real-world-weight cross-entropy loss, M is the number of training examples, $w_{\mathrm{mcfn}}$ is the marginal cost of a false negative, $w_{\mathrm{mcfp}}$ is the marginal cost of a false positive, $y_{m}$ is the target label for training example m, $x_{m}$ is the input for training example m, and $h_{\theta }$ is the model with neural network weights θ.^19^

## 3 Experimental Methods

### 3.1 Evaluation of Image Preprocessing

The contrast improvement index (CII) was calculated to quantify the contrast in the preprocessed image with respect to the input image.^24^^,^^25^ The CII was obtained by calculating the average local contrast C as measured in a 3×3 window swept over the image and dividing C for the proposed preprocessed image by C for the original image [Eqs. (2) and (3)]. A CII value >1 corresponds to an increase in image contrast

$$C=\frac{\max-\min}{\max+\min},$$ (2)

and

$$\mathrm{CII}=\frac{C_{\text{proposed}}}{C_{\text{original}}}.$$ (3)

### 3.2 Physician Image Quality Assessment

We conducted an image quality evaluation study to evaluate the effect of image preprocessing and harmonization. We surveyed a group of expert pediatric ophthalmologists, each having years of experience monitoring, imaging, diagnosing, and treating patients with ROP and plus disease. Physicians were presented with a graphical user interface (GUI) with images (with and without preprocessing), buttons to grade plus disease for each image, and a series of questions regarding their preference of the preprocessed or original images. Fifty images and their preprocessed counterparts were randomly selected from our image dataset and presented to the physicians in a randomized order, with each physician evaluating the same 100 images. Grading of the images occurred in three categories—“plus,” “no plus,” and “pre plus.” The physicians were presented with three questions after grading all 100 images, selecting from the responses “strongly disagree,” “disagree,” “neither agree nor disagree,” “agree,” and “strongly agree” on the 5-point Likert scale.

1. Did you find it easier to view the vessels in the processed images?
2. Will this image processing method improve your ability to diagnose plus disease?
3. Would you find it helpful to implement the processing method on your images immediately after acquisition?

We collected responses from eight pediatric ophthalmologists, two from the United States and six from Argentina. The physicians from the United States typically work with RetCam images, whereas those from Argentina use the smartphone as their primary tool for fundus image acquisition. It is important to note that, when making diagnoses on the GUI, physicians were forced to diagnose based only on the image displayed, whereas the ground truth diagnoses were made during an in-person clinical examination of the patient.

### 3.3 Deep Learning Classification Experiments

We conducted the following cross-validation experiments to investigate the consistency of our results:

1. stratified five-fold cross-validation experiment using cross entropy loss
2. stratified five-fold cross-validation experiment using RWWCE loss

and the following train/held-out test experiments to compare network performance to that of physicians:

1. train/test experiment on processed images using cross entropy loss
2. train/test experiment on processed images using RWWCE loss
3. train/test experiment on original (unprocessed) images using cross entropy loss.

The details on each of these experiments follow.

The stratified five-fold cross-validation experiments were conducted for four repetitions. For each repetition, the dataset was randomly split into five folds, with four splits constituting the training dataset and one the testing dataset (80% train and 20% test). The augmentation/randomized selection method was used to generate 2464 “no plus” images from 308 and 352 “plus” images from 44 and to create a balanced 600 image training dataset. The network performance was evaluated on the unaugmented testing dataset. This was performed for each of the five folds, and the experiment was repeated four times. The cross-validation experiment was conducted once using a standard cross-entropy loss function and again using the RWWCE loss function. The real-world cost, evaluated to compare loss function performance, was calculated as the cost of a false negative multiplied by the number of false negatives plus the cost of a false positive multiplied by the number of false positives, divided by the number of samples.^19^

The train/held-out test validation experiments, using the 50 images randomly selected for physician evaluation via the GUI as the held-out test set, were performed to analyze the relationship between physician performance and model performance. Each label from the remaining 390 images was split into training and validation sets (90% train and 10% validation). For the training set, the augmentation/randomized selection method was used to create a class-balanced training dataset of 600. We trained the network using standard cross-entropy loss and RWWCE loss. Training on the unprocessed images was then performed using the same methods, with the unprocessed versions of the same 50 images constituting the held-out test set, to evaluate the utility of the preprocessing method for the deep learning classifier.

### 3.4 Comparison Between Deep Learning and Physician Performance

We compared the performance of our preliminary deep learning model to that of the pediatric ophthalmologists. We again grouped the “no plus” category with the “pre plus” category and distinguished these from “plus,” as was done for the deep learning model, for the 50 image test set. Accuracy, sensitivity, and specificity were calculated for each physician evaluator, as well as for the deep learning model that performed best on the held-out test set.

## 4 Results

### 4.1 Evaluation of Image Preprocessing

**Fig. 3:**
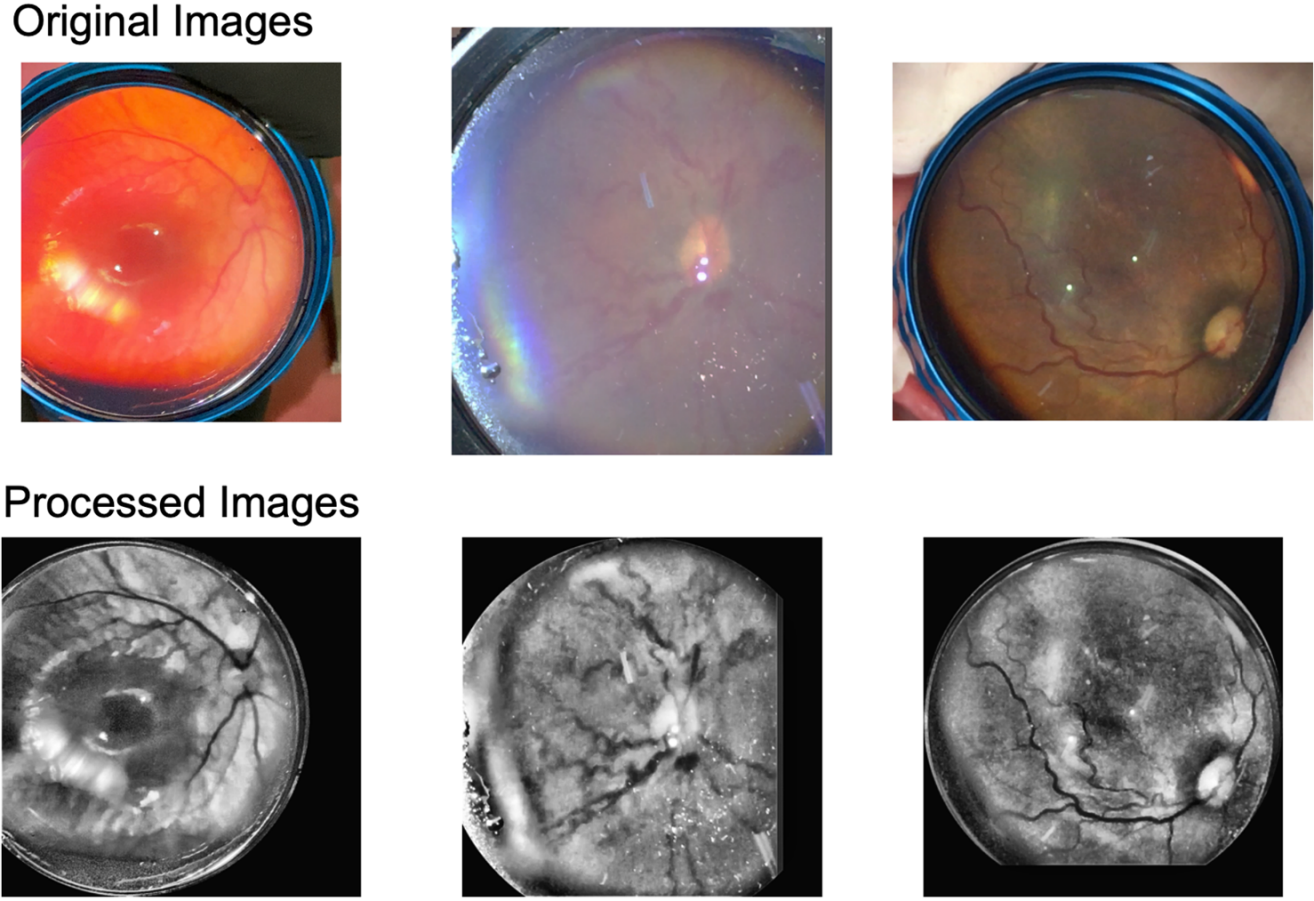
Original smartphone fundus images and corresponding images after preprocessing.

Image enhancement greatly improved the visibility and contrast of retinal vessels (Fig. 3). 10 out of the 440 total image pairs were randomly selected for evaluation of the CII. We found that the average CII for the subset of 10 images was 1.899, meaning the contrast was enhanced by 89.9% for the preprocessed images. Additionally, each image pair had a CII>1.

### 4.2 Physician Image Quality Assessment

**Fig. 4:**
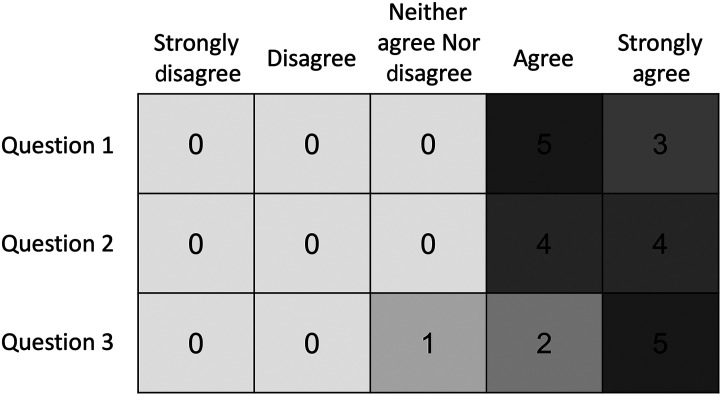
Responses of pediatric ophthalmologists to questions at the end of GUI. Question 1: Did you find it easier to view the vessels in the processed images? Question 2: Will this image processing method improve your ability to diagnose plus disease? Question 3: Would you find it helpful to implement the processing method on your images immediately after acquisition?

Responses from the physician image quality experiment are recorded in Fig. 4. Physicians answered “agree” or “strongly agree” to every question, with the exception of one participant responding “neither agree nor disagree” to the third question, “Would you find it helpful to implement the processing method on your images immediately after acquisition?” It should be noted that this physician practices in the United States and thus does not use smartphone images for diagnosis, so there may have been some ambiguity in this question.

### 4.3 Deep Learning Classification Experiments

**Fig. 5:**
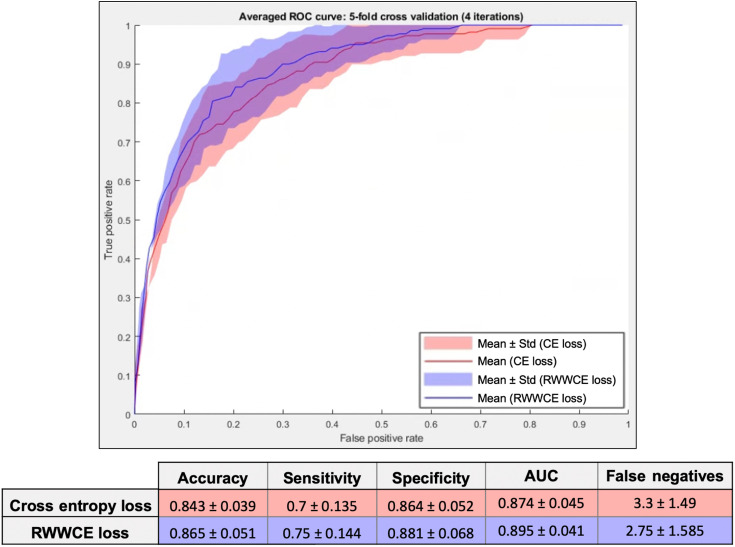
ROC curves for five-fold cross validation, with four iterations, using both the cross entropy loss and the real-world weight cross-entropy loss.

We performed cross-validation experiments using both the standard cross-entropy loss function and the RWWCE loss function. ROC curves were averaged over all 20 iterations (5 folds with 4 repetitions) and their standard deviations were calculated. Averaged ROC curves and standard deviations are shown in Fig. 5, along with traditional metrics (accuracy, sensitivity, specificity, and AUC) averaged over 20 iterations. Accuracy, sensitivity, and specificity were measured using a threshold of 0.5. Using the RWWCE loss function, the average number of false negatives over 20 iterations (the number of infants with plus disease that would be misclassified and thus not treated) becomes 2.65 from a previous value of 3.3 using the cross-entropy loss function. To understand this, consider a cohort of 1000 infants with a prevalence of plus disease of 12.5%, as in our dataset. Using smartphone images for diagnosis, based on the network trained using cross-entropy loss, 38 infants with plus disease would be misdiagnosed, whereas based on the network trained using RWWCE loss, 30 infants having plus disease would be misdiagnosed. Although this may seem like a small numerical improvement, aside from eight babies not going blind being the biggest gain, when considering the weighting of the cost of a false negative at $1,700,000, or what we have determined to be the “cost of lifelong blindness,” this is a change of $13,600,000. Furthermore, the average real-world cost using the cross-entropy loss function was $65,768.75 over the 20 iterations of test sets, whereas that using the RWWCE loss function was $54,892.61. Our results demonstrate the benefit of employing the RWWCE loss function in cases in which the cost of missing a case of the disease vastly outweighs that of falsely identifying the disease.

**Fig. 6:**
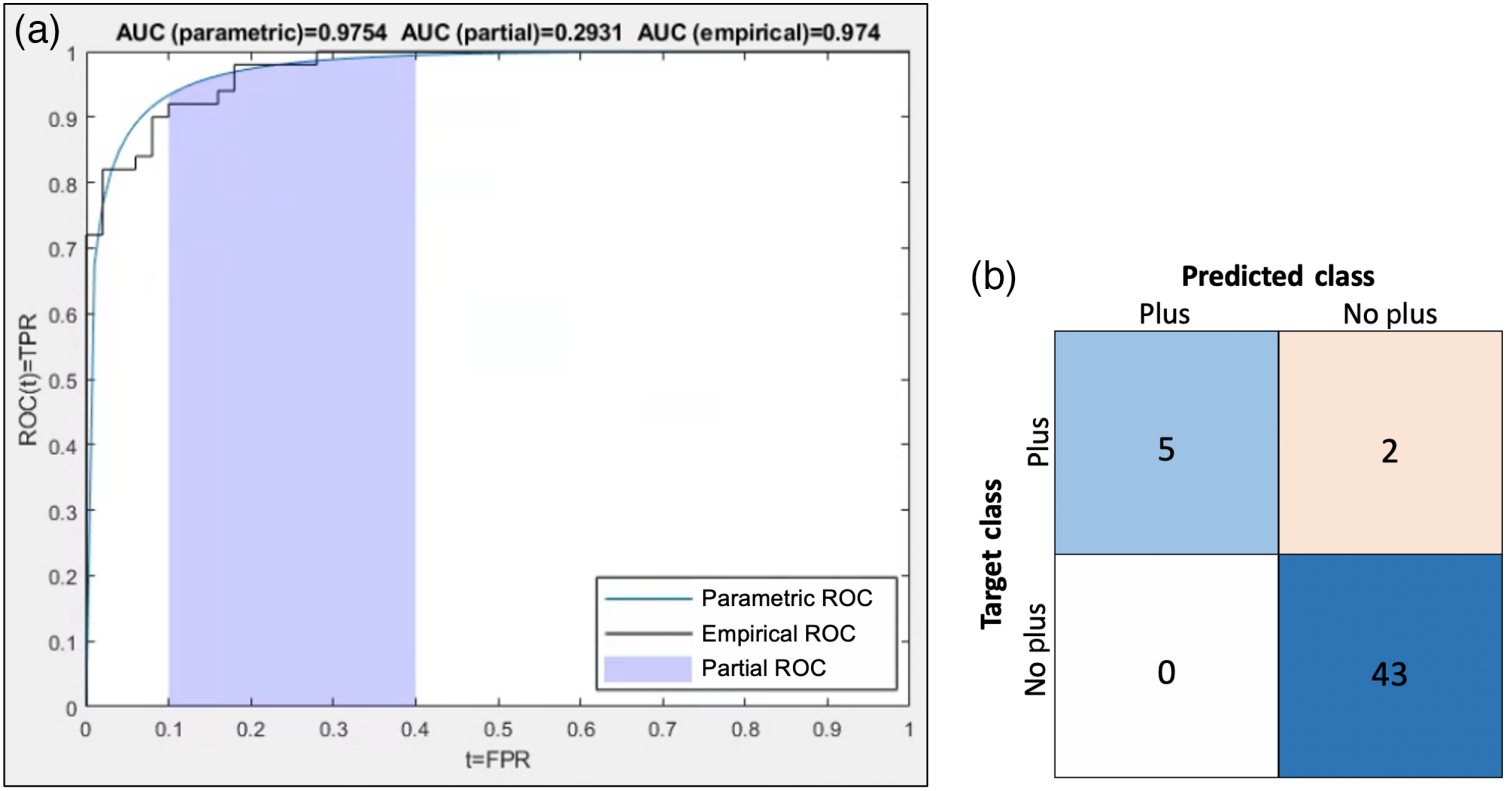
(a) ROC curve, including both parametric and empirical estimations and (b) confusion matrix for the test set.

**Fig. 7:**
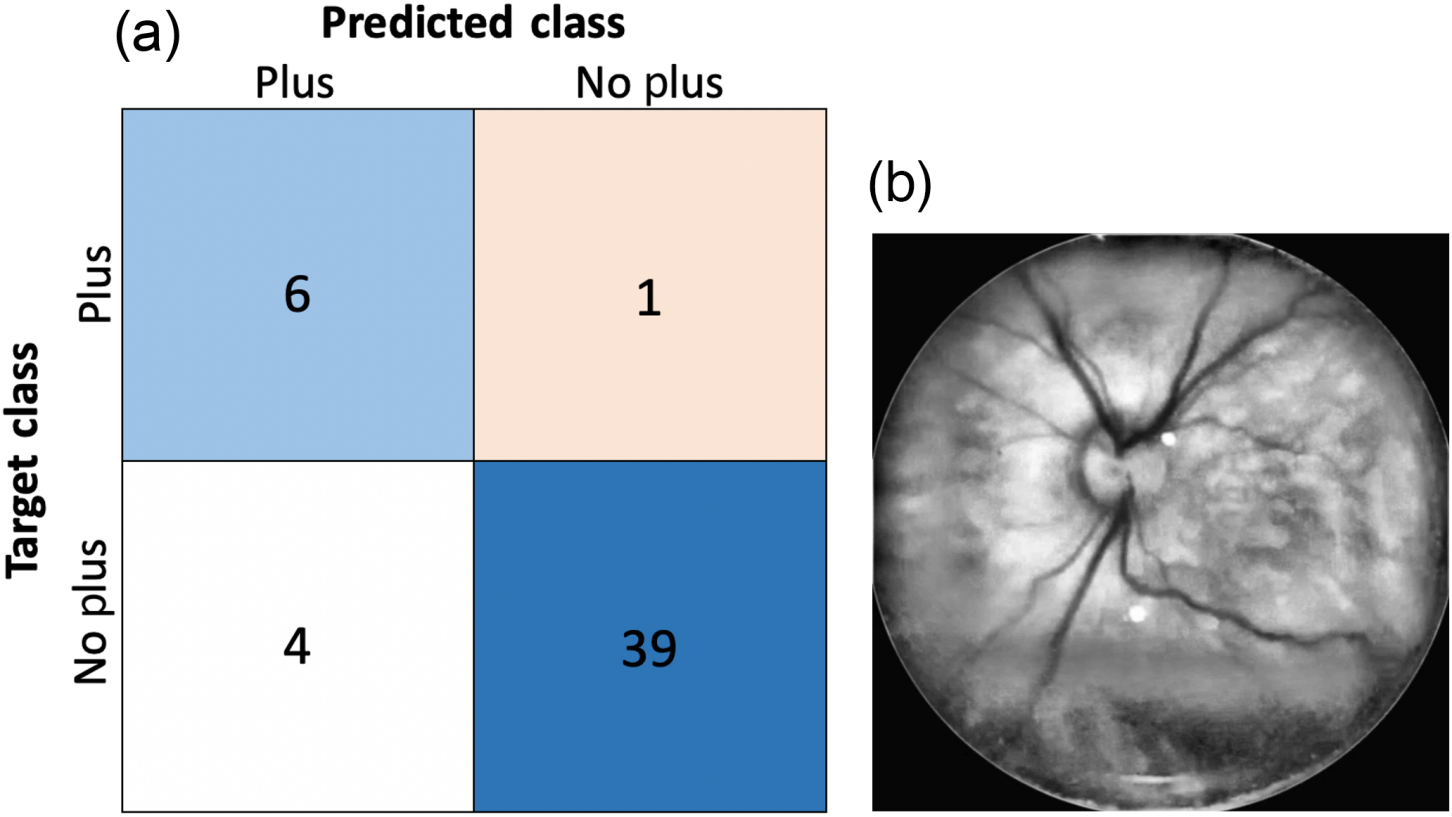
Results on the held-out test set using the real-world-weighted cross-entropy loss function. (a) Confusion matrix for held-out test set and (b) the inaccurately classified plus disease image.

**Fig. 8:**
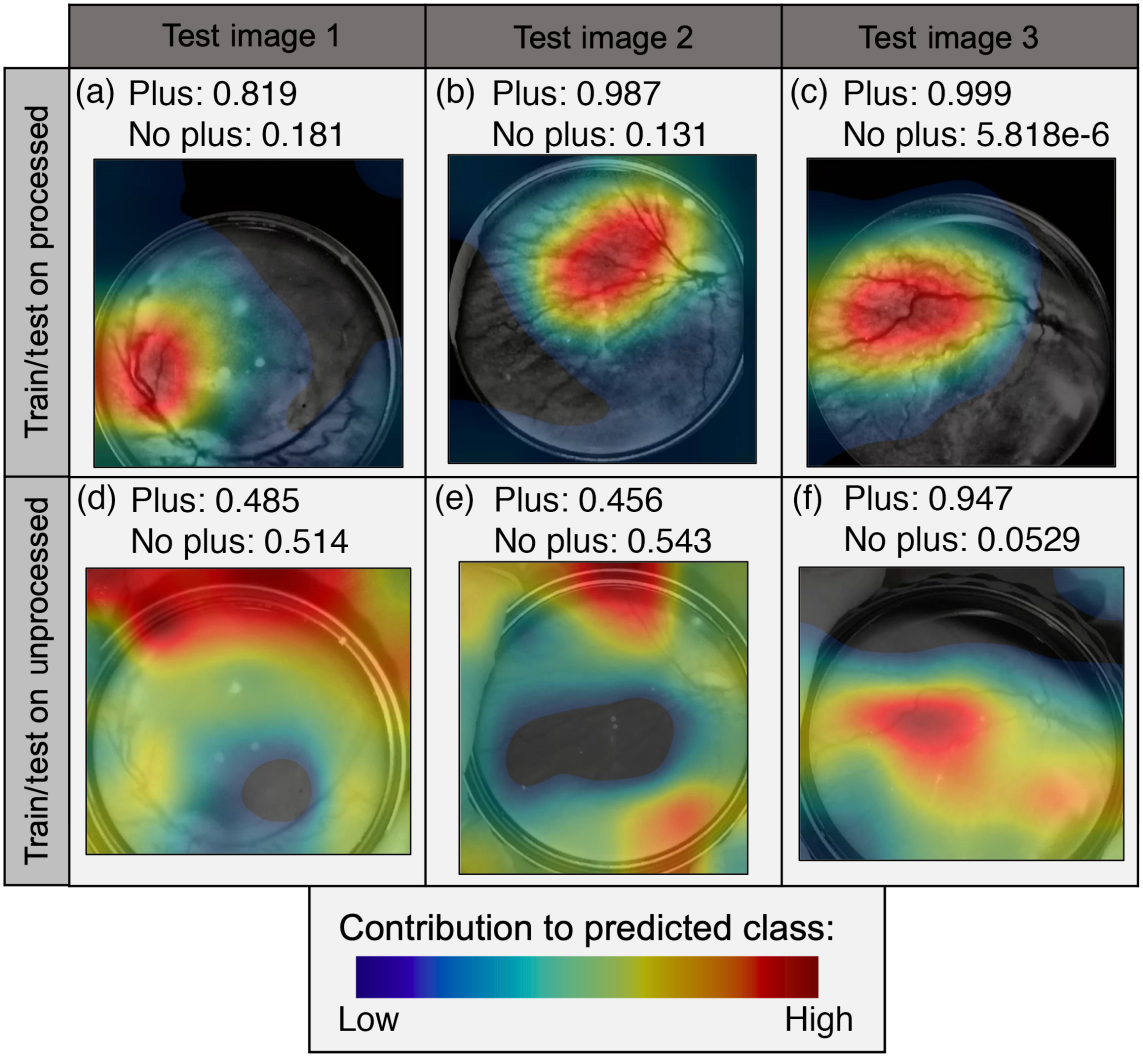
Class activation maps. (a)–(c) Test images when network training/testing uses a processed dataset. (d)–(f) Corresponding images when network training/testing uses the original unprocessed dataset.

On the held-out test set, training/testing on the preprocessed images gave promising results, and doing so on the unprocessed images provided insight into the utility of the preprocessing method. When training/testing using the preprocessed images, we obtained an AUC of 0.9754, accuracy of 0.96, specificity of 1, and sensitivity of 0.7143 on the held-out test set of 50 images. The precision was 1, recall was 0.7143, and the F1 score was 0.833. The ROC plot shows a high AUC for both empirical and parametric estimations of the ROC curve, and two of the seven “plus” images were classified as “no plus” in the confusion matrix (Fig. 6).^26^ Results using the RWWCE loss function for the train/held-out test experiments on the preprocessed images are reported in Fig. 7. The loss function correctly identified an additional “plus” case at the expense of four false positives. We finally evaluated deep learning classification on images without preprocessing, using the standard cross-entropy loss function. Classifier performance was not quantifiably much worse than in the corresponding experiment using processed images, also only misclassifying two of seven “plus” cases and accumulating five false positives. However, the assessment of the class activation maps for both the network trained/tested on the original and preprocessed images demonstrates that, without processing, the network relies on unreasonable regions of the image to make classification decisions (Fig. 8). Class activation maps were generated using a weighted sum of the feature maps of the final convolutional layer and highlighted discriminative regions critical to the final classification of the image.^27^ It is clear from the activation maps that the network is focusing on haphazard regions of the unprocessed images for classification, but after preprocessing, it focuses on a targeted region of the vasculature.

### 4.4 Comparison Between Deep Learning and Physician Performance

**Fig. 9:**
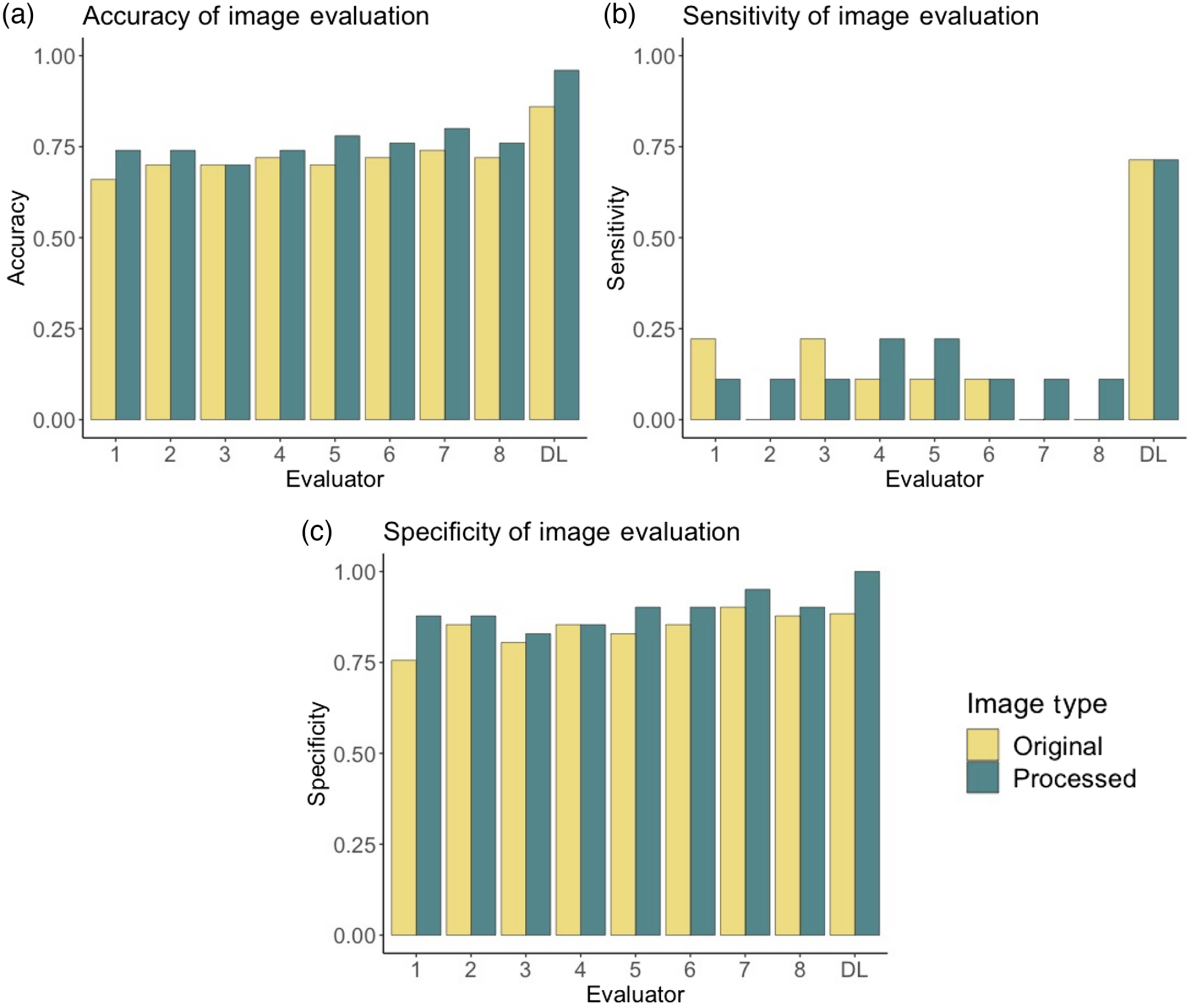
Comparative performances of physicians and deep learning on a held-out test set. (a) Accuracy, (b) sensitivity, and (c) specificity.

As seen in Fig. 9, the deep learning classifier outperformed the physicians in terms of accuracy, sensitivity, and specificity for a binary plus disease diagnosis. Sensitivity is particularly of interest as it corresponds to the number of correctly classified “plus” cases, for which deep learning largely outperformed the ophthalmologists. In 91.7% of cases, the physician performance on preprocessed images was better than or equal to their performance on the original images, demonstrating the utility of the preprocessing method for physician use.^28^ The accuracy and sensitivity of classification by physicians were both statistically greater on preprocessed images than on the original images, as determined using one-sided paired t-tests (p=0.00127 and p=0.00579, respectively).

## 5 Discussion

To address the unmet need for accurate, affordable access to ROP staging around the world, we investigated preprocessing enhancements and AI on fundus images obtained using a simple handheld lens and a smartphone. Preprocessing increased vessel visibility and harmonized smartphone images as evaluated by physicians. Remarkably, deep learning classification on single images gave very good classification results compared to a ground truth from physicians examining the infant in-person with consideration of the patient’s condition. Furthermore, deep learning outperformed physicians making diagnoses based on single smartphone images alone (Fig. 9). Although deep learning on unprocessed images gave similar classification results to that on preprocessed images, class activation maps suggested that deep learning with unprocessed images focused on clinically unimportant image regions as compared with a network trained with the preprocessed images that we developed (Fig. 7). Activation map analysis suggests that preprocessing might give more generalizable results across a larger variety of images. Cell phone images certainly have limitations, but our image enhancement steps greatly improve their utility. The image size is regularized, the contrast of vessels is greatly enhanced, and images can be read in sub-par lighting conditions. Image enhancement was preferred by pediatric ophthalmologists for decision making, quantitatively increased contrast and vessel visibility, and facilitated the development of a deep learning model with high accuracy and explainability.

Image enhancement and harmonization proved beneficial not only for physician evaluation but also for deep learning classification. Class activation maps on preprocessed images suggest that the network classifies images as “plus” by looking at the first branching after the optic nerve and classifies “no plus” based on the region immediately surrounding the optic nerve. This is in contrast to physicians, who examine the entirety of the infant’s eye, including regions out of the field of view of these images, and make final decisions based on the optic nerve alone. This is interesting moving forward as we continue to develop software for automated plus disease diagnosis and potentially incorporate peripheral fundus images into our work.

We found that, when evaluated on the same held-out test set presented to trained ophthalmologists for grading, the deep learning model greatly outperformed physicians, particularly in terms of sensitivity (Fig. 9). This is likely due to the fact that ophthalmologists are accustomed to diagnosis based on an in-person clinical eye exam or a gold-standard RetCam image. The successful performance of our deep learning solution suggests the potential for accurate diagnosis using smartphone fundus images and the future utilization of these images for telemedicine-based diagnoses.

Our project had some limitations. First, we had access only to a limited dataset of 440 images. Second, we selected single images for deep learning work. Our selection criteria for images included in our study was the presence of the optic nerve; however, inclusion of additional images showing peripheral anatomy might provide a more complete picture for network analysis. Despite the limited number of cases of plus disease in our dataset, we developed an experimental protocol to measure the physicians’ abilities to use low quality, low field of view smartphone fundus images to diagnose plus disease and compare this with the deep learning performance. Future work will involve further collaboration with other countries in the SP-ROP project and access to more smartphone images.

## 6 Conclusion

We have demonstrated the ability to enhance cell phone images of the fundus for improved visualization and the ability to detect plus disease using AI. Results suggest that it might be possible to create local or cloud software to improve the diagnosis of plus disease in low-resource settings, saving many premature infants from blindness. We hope that our work can stimulate further efforts for creative low-cost solutions to this critical issue. With accessible, accurate staging, clinicians can treat the right patients and reduce the rates of the leading cause of preventable blindness in children.

## Data Availability

The data that support the findings of this article are not publicly available due to ethical concerns, as they require a data use agreement. They can be requested from the SP-ROP group by contacting Guillermo Monteoliva. The code used in this work can be accessed through Code Ocean: https://codeocean.com/capsule/9799545/tree.
